# Temporal evolution of the resistance genotypes of *Plasmodium falciparum* in isolates from Equatorial Guinea during 20 years (1999 to 2019)

**DOI:** 10.1186/s12936-021-04000-w

**Published:** 2021-12-14

**Authors:** Pedro Berzosa, Irene Molina de la Fuente, Thuy-Huong Ta-Tang, Vicenta González, Luz García, Ana Rodríguez-Galet, Ramón Díaz-Regañón, Rosario Galán, Laura Cerrada-Gálvez, Policarpo Ncogo, Matilde Riloha, Agustin Benito

**Affiliations:** 1 National Centre of Tropical Medicine-Institute of Health Carlos III, Madrid, Spain; 2 Department of Biomedicine and Biotechnology, University of Alcalá and National Centre of Tropical Medicine-Institute of Health Carlos III, Madrid, Spain; 3 HIV Molecular Epidemiology Laboratory, Ramón y Cajal-IRyCIS Hospital, Madrid, Spain; 4 State Foundation, Health, Childhood and Social Welfare FSP, Madrid, Spain; 5 Ministry of Health and Social Welfare-Malaria National Programme of Equatorial Guinea, Malabo, Equatorial Guinea

**Keywords:** Malaria, Resistance, Genes, Artemisinin combination therapy, Equatorial Guinea

## Abstract

**Background:**

Malaria is one of the deadliest diseases in the world, particularly in Africa. As such, resistance to anti-malarial drugs is one of the most important problems in terms of global malaria control. This study assesses the evolution of the different resistance markers over time and the possible influence of interventions and treatment changes that have been made in Equatorial Guinea.

**Methods:**

A total of 1223 biological samples obtained in the period 1999 to 2019 were included in the study. Screening for mutations in the *pfdhfr*, *pfdhps*, *pfmdr1,* and *pfcrt* genes was carried out by nested PCR and restriction-fragment length polymorphisms (RFLPs), and the study of *pfk13* genes was carried out by nested PCR, followed by sequencing to determine the presence of mutations.

**Results:**

The partially and fully resistant haplotypes (*pfdhfr* + *pfdhps*) were found to increase over time. Moreover, in 2019, the fully resistant haplotype was found to be increasing, although its super-resistant counterpart remains much less prevalent. A continued decline in *pfmdr1* and *pfcrt* gene mutations over time was also found. The number of mutations detected in *pfk13* has increased since 2008, when artemisinin-based combination therapy (ACT) were first introduced, with more mutations being observed in 2019, with two synonymous and five non-synonymous mutations being detected, although these are not related to resistance to ACT. In addition, the non-synonymous A578S mutation, which is the most frequent on the African continent, was detected in 2013, although not in the following years.

**Conclusions:**

Withdrawal of the use of chloroquine (CQ) as a treatment in Equatorial Guinea has been shown to be effective over time, as wild-type parasite populations outnumber mutant populations. The upward trend observed in sulfadoxine-pyrimethamine (SP) resistance markers suggest its misuse, either alone or in combination with artesunate (AS) or amodiaquine (AQ), in some areas of the country, as was found in a previous study conducted by this group, which allows selective pressure from SP to continue. Single nucleotide polymorphisms (SNPs) 540E and 581G do not exceed the limit of 50 and 10%, respectively, thus meaning that SP is still effective as an intermittent preventive treatment (IPT) in this country. As for the *pfk13* gene, no mutations have been detected in relation to resistance to ACT. However, in 2019 there is a greater accumulation of non-synonymous mutations compared to years prior to 2008.

**Graphical Abstract:**

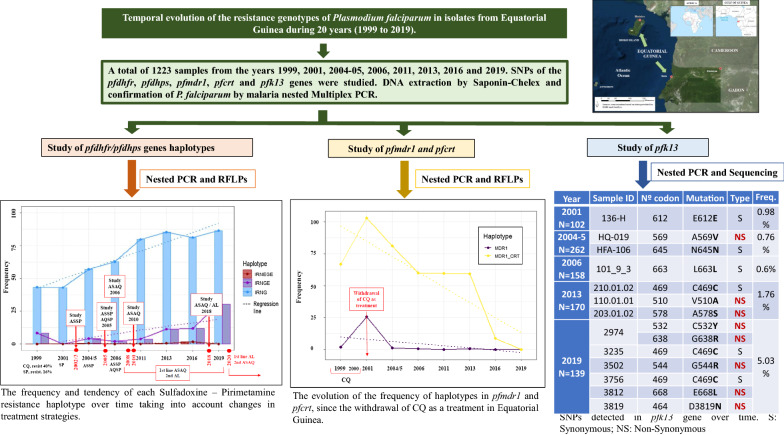

## Background

Although malaria control has increased significantly worldwide, this parasitic disease remains one of the deadliest in the world, particularly in Africa, where 85% of fatal cases occur. Indeed, an estimated 229 million cases of malaria and 409,000 deaths were recorded in 2019 [1]. In Equatorial Guinea, a country located in West Central Africa, *Plasmodium* infections are one of the leading causes of disease, with an incidence rate of 352,124 cases per year, and they are responsible for 15% of deaths among children under the age of 5 years.

The main strategy for malaria control is quick and accurate diagnosis followed by effective treatment [2]. To ensure the efficacy of the treatment, therapeutic efficacy studies of first- and second-line anti-malarial treatments should be carried out at least once every 2 years, as recommended in the World Health Organization (WHO) standard protocol for monitoring drug efficacy [3]. This provides confirmation that the treatment continues to work properly and the patient is guaranteed to receive quality treatment. In 2009, Equatorial Guinea adopted artemisinin-based combination therapy (ACT), specifically artesunate-amodiaquine (ASAQ), as first-line treatment [4, 5]. However, after the latest therapeutic efficacy study carried out in that country [6], the first-line treatment for uncomplicated malaria was changed from ASAQ to artemether/lumefantrine (AL) [7].

The emergence of drug resistance, particularly among *Plasmodium falciparum* parasites, the most prevalent species in the country, has been a major contributor to the global burden of malaria in the past 30 years [8]. Indeed, resistance is the most likely explanation for the doubling of malaria-related child deaths in eastern and southern Africa [9].

Similarly, the spread of anti-malarial resistance in *P. falciparum* parasites has also been a major obstacle to global malaria control and eradication [10]. The emergence of resistance to chloroquine (CQ) led to its substitution by sulfadoxine/pyrimethamine (SP), which was widely introduced for the treatment of uncomplicated *P. falciparum* malaria [11], although an increase in parasite resistance to SP subsequently occurred. Nowadays, although is not useful as a treatment in Africa due to widespread drug resistance, it is routinely implemented as an intermittent preventive treatment (IPT) for malaria, particularly during pregnancy (IPTp) and in infants (IPTi) [12, 13]. Given the importance of the use of IPT for disease prevention in the most vulnerable groups, it would be of particular use to determine the temporal evolution of resistance to SP [14]. In this context, surveillance of molecular resistance markers plays a key role in the decision-making process for malaria control.

Resistance to SP has been associated with a single nucleotide polymorphism (SNP) in two different genes, namely the dihydrofolate reductase (*pfdhfr*) and dihydropteroate synthase (*pfdhps*) genes, which encode for the enzymes PfDHFR and PfDHPS, respectively, both of which are important in the folate synthesis pathway [15, 16]. N51I, C59R, S108N, and I164L mutations in the *pfdhfr* gene confer pyrimethamine resistance, and A437G, K540E and A581G mutations in the *pfdhps* gene confer sulfadoxine resistance [17]. Naidoo et al. have described three combinations of SNPs related to SP resistance: partially resistant (quadruple mutant: *pfdhfr* 51I59R/108N + *pfdhps* 437G), IRNG haplotype; fully resistant (quintuple mutant: *pfdhfr* 51I/59R/108N + *pfdhps* 437G/540E), IRNGE haplotype; and, super resistant (sextuple mutant: *pfdhfr* 51I/59R/108N + *pfdhps* 437G/540E/581G), IRNGEG haplotype [18].

The K76T mutation in the CQ-resistant transporter gene (*pfcrt*) has been associated with AQ and CQ resistance. Indeed, different studies carried out in sub-Saharan African countries, such as south eastern Cameroon [19], Kenya and Malawi, have suggested that the withdrawal of CQ pressure from the population led to a gradual reduction in the proportion of circulating mutant genotypes of the *Pfcrt* gene, thereby increasing the wild genotypes in the population [20–23]. Moreover, SNPs at positions N86Y and D1246Y in *pfmdr1* (*P. falciparum* multi-drug resistance gene) were associated with modulated parasite tolerance and susceptibility to a number of anti-malarial drugs, including quinine, amodiaquine (AQ), CQ (although it plays a secondary role here), mefloquine (MQ), and lumefantrine (L) [24]. Furthermore, amplifications of the *pfmdr1* gene may cause resistance to artesunate (AS), and there is some evidence that AQ use may induce selection of the *pfcrt* T76 and *pfmdr1* Y86 mutant alleles [25]. This finding may provide some insight into the cross-resistance observed between CQ and AQ in vivo. Mutant *pfcrt* T76 and *pfmdr1* Y86 alleles, which are currently used as molecular markers for CQ resistance, may also be useful for monitoring the spread of AQ resistance, such as in West Africa [24].

With regard to the resistance of *P. falciparum* to artemisinin (ART) and its derivatives, this has been widely documented in Southeast Asia (SEA) [26]. As such, the recent gains in global malaria control as a result of ACT is threatened by the emergence of artemisinin resistance in SEA and the probable spread to sensitive areas [27]. However, comparatively speaking, only low-level ART resistance has been identified in Africa to date [28]. Despite this, ongoing worldwide surveillance is still necessary due to the potential public-health impact such resistance could have, especially in children under 5 years of age and pregnant women from Africa, as well as non-immune travellers [28]. ART resistance in the SEA region has been linked to the kelch propeller domain on chromosome 13 (*pfk13*) [29, 30], therefore, SNPs, and consequent amino acid changes in the gene that occurred because of drug pressure are typically used as molecular markers. The *pfk13* resistance markers are non-synonymous mutations and include F446I, N458Y, M476I, Y493H, R539T, I543T, P553L, R561H, P574L, and C580Y [31, 32] as validated markers. The principal mutations related to resistance are C580Y, Y493H, R539T, I543T, and N458Y, which were observed in all isolates with the slow-clearance phenotypic trait. The principal mutations associated with resistance observed in African isolates are C580Y, seen in Cameroon, and Y493H, seen in Ghana. Other mutations observed in ART-resistant parasites from the SEA region, which have also been described in African isolates, include S522C, P553L, R561H, A675V, and H719N [33]. The other predominant *pfk13* non-synonymous mutation found in African isolates is A578S, which has been observed in all African countries in which *pfk13* mutations have been typed, although there is no phenotypic association with ART resistance [34]. However, since it is close to the validated mutation C580Y, which is known to be related to resistance, it is thought that A578S may be the mutant for ART resistance in African isolates [35, 36] even though there is no phenotypic association with ART resistance. Of all the *pfk13* mutations observed only in African isolates, and not in SEA isolates, none has yet been directly linked to ART resistance; therefore, further studies are still needed [32, 37].

The objectives of this study were to determine the temporal trends in the prevalence of multi-locus anti-malarial resistance markers in *P. falciparum* isolates collected from clinical sources in Equatorial Guinea between 1999 and 2019. This includes analysis of the evolution of markers associated with SP resistance (*pfdhfr* and *pfdhps*), AQ and CQ resistances (*pfmdr1* and *pfcrt)* and artemisinin resistance (*pfk13)*. This study also offers the opportunity to observe the evolution of the different resistance markers over time and the possible influence of the different interventions and treatment changes made in the country.

## Methods

### Biological samples and location

**Fig. 1:**
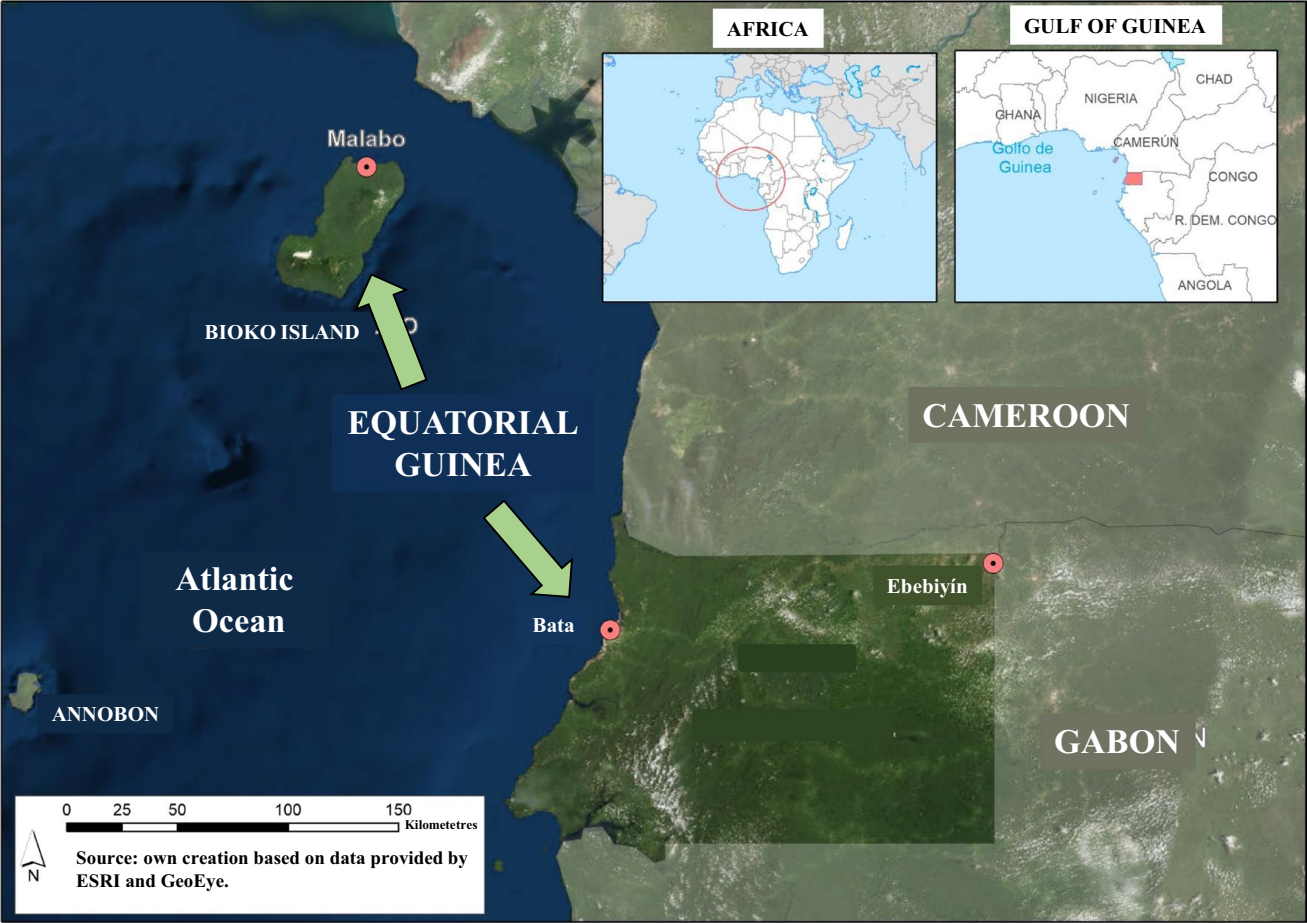
Map showing the location of Equatorial Guinea in Africa. The small boxes show the location of Equatorial Guinea on the African continent. The two regions of the country are also indicated, the Island Region (Bioko Island where is the capital city and Annobon) and the Continental Region between Cameroon and Gabon

Equatorial Guinea is located in West Central Africa. It is divided into an Insular Region (Bioko Island, where the capital city, Malabo, is located, and Anobón, Elobey and Corisco), with the Continental Region lying between Cameroon and Gabon (Fig. 1). All samples used in this study come from the biological samples collection of the National Centre of Tropical Medicine, which is registered with the National Biobank at the Institute of Health Carlos III (Madrid, Spain) in accordance with Spanish Law RD 1716/2011 (registration number C0005278). The samples selected were obtained between 1999 and 2019 as part of different studies carried out in Equatorial Guinea in collaboration with the National Malaria Control Programme of the Ministry of Health and Social Welfare. Table 1 shows the different efficacy studies carried out between 1990–92 and 2019, along with the different changes in treatment in that country and the year of the samples analysed in the study.

**Table 1:** Treatments and efficacy studies carried out over time in Equatorial Guinea

Years	Treatments and efficacy studies	No of Samples
1990–92	In vitro susceptibility of *P. falciparum* to CQ, AQ, MQ and SP in Equatorial Guinea [44]	
1996*	Resistance: 55% CQ	
1999*	Use of CQ as treatment. An efficacy study was conducted and a resistance of 40% was found (16% to SP) [45]. CQ was withdrawn	N = 60
2001*	SP introduced as a treatment	N = 102
2002/2003	Study of the efficacy of the combination AS-SP: 95% efficacy (unpublished study)	
2004*	AS-SP introduced	N = 262
2005*	Study of the efficacy of the combinations AS-SP and AQ-SP [4]	
2006*	ASSP and AQSP Efficacy study of ASAQ [5]	N = 158
2008	Change of official treatment: 1st line ASAQ, 2nd line AL	
2010	Efficacy study for ASAQ (unpublished study): 95% efficacy	
2011*	1st line ASAQ, 2nd line AL	N = 163
2013*	1st line ASAQ, 2nd line AL	N = 170
2016*	1st line ASAQ, 2nd line AL	N = 169
2018	1st line ASAQ, 2nd line AL Efficacy study of ASAQ and AL [6]	
2019*	1st line ASAQ, 2nd line AL	N = 139
2020	Change of official treatment: 1st line AL and 2nd line ASAQ	

An asterisk (*) indicates the years from which samples were taken for the National Centre for Tropical Medicine collection. The different efficacy studies that have been carried out in Equatorial Guinea between 1990–92 and 2020. How the treatments changed in each year is indicated. The ‘samples’ column indicates the number of samples that were analysed for each year included in the study

CQ: Chloroquine; AQ: amodiaquine; MQ: mefloquine; SP: sulfadoxine/pyrimethamine; AS: artesunate; L: lumefantrine; A: artemether

### DNA extraction and PCR amplification

The samples in the collection are dried finger blood on Whatman 903™ filter paper (GE Healthcare Bio-Sciences Corp.) from patients with malaria diagnosed by microscopy and confirmed by nested multiplex malaria PCR (NM-PCR) [38, 39]. All samples were stored at − 20 °C. Samples diagnosed as *P. falciparum*, were selected to screen the mutations related to drug resistance in the *P. falciparum* genes *pfdhfr*, *pfdhps*, *pfmdr1*, *pfcrt* and *pfk13.*

#### DNA extraction

DNA was extracted from the above samples using the Saponin/Chelex method [40] with minor modifications for our laboratory. Thus, a 5-mm diameter punch containing 10 μL of blood was used. The tube containing the isolated DNA was then labelled with the sample number, year and place of origin of the sample. These DNA samples were used immediately for PCR or stored at − 20 °C until use.

#### Nested PCR molecular markers for resistance

The *P. falciparum* genes *pfdhfr*, *pfdhps*, *pfmdr1*, *pfcrt* and *pfk13* were analysed*.* Mutation screening was performed as described in Maryland University Protocols by Plowe et al. [41], with minor modifications. The nested PCR used produces amplification fragments for each of the genes under study, *pfdhfr*, *pfdhps*, *pfmdr1* and *pfcrt*. Each fragment includes the following codon positions that are studied: 108, 164, 51, and 59 in *pfdhfr*; 437, 540 and 581 in *pfdhps*; 86 and 1246 in *pfmdr1* and 76 in *pfcrt.* PCR products were separated by electrophoresis on a 2% agarose gel, stained with Pronasafe (Pronadisa, Spain), identified based on the fragment size and visualized under an ultraviolet transilluminator. The amplification fragments were digested with different restriction enzymes to analyse restriction-fragment length polymorphisms (RFLPs). Each mutation point in each of the genes requires a different enzyme (New England BiolabsR Inc.) to determine whether or not there is a mutation at that position. Each such enzyme was used according to the manufacturer’s recommendations. The haplotypes of *pfdhfr* and *pfdhps* gene haplotypes were classified as partially resistant (IRNG), fully resistant (IRNGE) or super resistant (IRNGEG) [18]. The haplotypes of the other genes studied comprised one double mutation in a single gene: 86Y/1246Y *pfmdr1* and a combination of two single mutations in different genes: 86Y *pfmdr1* + 76T *pfcrt*.

#### Nested PCR and sequencing of the *pfk13* gene

The nested PCR protocol described by Ariey et al. [29, 42] was used, with some modifications. Polymerase HotStart (5 U/mL) (Biotools, Spain), at a final concentration of 0.028 U/mL, was used to standardize this PCR protocol. For the first PCR, 5 mL of genomic DNA was used, along with 0.25 mM (final concentration) of each primer: **K13-PCR-F** (5′-GGGAATCTGGTGGTAACAGC-3′)/**K13-PCR-R** (5′-CGGAGTGACCAAATCTGGGA-3′). The volume of the PCR mix was 25 mL. The second PCR (nested) was performed with 0.25 mM (final concentration) of each primer: **K13-N1-F **(**5**′**-**GCCTTGTTGAAAGAAGCAGA-3′)**/K13-N1-R **(**5**′**-**GCCAAGCTGCCATTCATTTG-3′), with a final volume of the PCR mix of 50 mL. After the second PCR, an electrophoresis on 2% agarose gel stained with Pronasafe (Pronadisa, Spain) was carried out. The estimated size of the amplification fragment is ± 850 bp.

The PCR products were purified using Ilustra exoprostar 1-step (GE Healthcare Life Sciences) in accordance with the manufacturer’s instructions. Samples were sequenced from both directions using the forward and reverse primers in the second PCR (K13-N1-F/ K13-N1-R) at a concentration of 6 pmol/mL, using a standard dye terminator (Big Dye Terminator v3.1 Cycle Sequencing kit) in an ABI PRISM 3730 XL Analyser. Sequences were compared with the Genebank database using BLAST (Basic Local Alignment Search Tool) [43], to check that the *pfk13* gene had been correctly sequenced.

To find new mutations and validated mutations in the *pfk13* gene related to resistance to artemisinin derivatives, the sequence of *Pfk13* gene 3D7 clone (PF3D7_1343700 kelch protein propeller domain) was compared with all the sequences obtained from each sample using BioEdit 7.2 software.

### Data analysis

The prevalence of mutations and haplotypes was calculated for each group of samples, and changes in the prevalence of mutations and haplotypes over time were compared using X^2^ statistics or Fisher’s Exact test, as appropriate. Logistic regressions were plotted to determine temporal trends in haplotypes, with the statistical significance being assessed using the Mann–Kendall trend test. The odds ratio (OR) was used to represent the relative changes between sample groups for the different years included in the study. All statistical tests were performed at a significance level of 5% (p-value < 0.05) and 95% confidence interval (CI). Statistical data analysis was conducted using the R 4.0.0 software package.

## Results

A total of 1223 samples obtained from patients with uncomplicated malaria between 1999 and 2019 were analysed in this study: 1999 (n = 60), 2001 (n = 102), 2004/5 (n = 262), 2006 (n = 158), 2011 (n = 163), 2013 (n = 170), 2016 (n = 169) and 2019 (n = 139). All samples were positive for *P. falciparum* by NM-PCR, and no mixed infection was detected.

### *pfdhfr* and *pfdhps* genes study

#### SNPs in *Pfdhfr* and *pfdhps* genes

Considering SNPs individually, it was observed that the frequency of the most prevalent mutations in ***pfdhfr***, namely 51I, 59R and 108N, increased from 96.6, 93.3 and 93.3% in 1999, to 97.1, 94.2 and 100%, respectively, in 2019. These mutations are approaching fixation in the population. Significant differences were detected for *pfdhfr* mutations when comparing the increase in the frequencies of the mutation at position 108N between 1999 and 2019 (p = 0.002). The frequencies of these SNPs were found to increase over time when the frequencies of all years included in the study were taken into account, with significant differences being observed (Table 2).

**Table 2:** SNPs detected in *pfdhfr* and *pfdhps* genes over the years

Year (N)	*Pfdhfr* gene SNPs						*Pfdhps* gene SNPs					
	51I		59R		108 N		437G		540E		581G	
	N (%)	95% CI	N (%)	95% CI	N (%)	95% CI	N (%)	95% CI	N (%)	95% CI	N (%)	95%CI
1999 (N = 60)	51 (58%)	88.64–99.08	56 (93.3%)	84.07–97.38	56 (93.3%)	88.64–99.08	30 (50%)	37.74–62.24	4 (6.6%)	2.62–15.93	3 (5%)	1.71–13-70
2001 (N = 102)	95 (93.1%)	86.51–96.64	88 (86.3%)	78.27–91.64	83 (81.4%)	72.73–87.74	62 (60.7%)	51.08–69.70	0	0–3.63	0	0–3.63
2004/5 (N = 262)	255 (93.3%)	94.59–98.70	259 (98.8%)	96.69–99.61	257 (98.1%)	95.61–99.18	158 (60.3%)	54.27–66.04	0	0–3.63	0	0–3.63
2006 (N = 158)	150 (94.9%)	90.33–97.41	151 (95.5%)	91.14–97.84	158 (100%)	97.63–100	44 (27.8%)	21.45–35.3	4 (2.5%)	0.99–6.33	0	0–2.37
2011 (N = 163)	159 (97.5%)	93.86–99.04	159 (97.5%)	93.86–99.04	163 (100%)	97.7–100	136 (93.4%)	76.97–88.36	9 (5.5%)	2.93–10.16	3 (1.8%)	0.63–5.27
2013 (N = 170)	168 (98.8%)	95.81–99.68	167 (98.2%)	94.94–99.4	168 (98.8%)	95.81–99.68	149 (87.6%)	81.85–91.78	26 (15.3%)	10.66–21.47	1 (0.58%)	0.10–3.26
2016 (N = 169)	169 (100%)	97.78–100	149 (88.2%)	82.43–92.21	168 (98.8%)	95.81–99.68	156 (92.3%)	87.29–95.45	23 (13.6%)	9.24–19.59	5 (3%)	1.27–6.74
2019 (N = 139)	135 (97.1%)	92.83–98.88	131 (94.2%)	89.05–97.06	139 (100%)	97.31–100	130 (93.5%)	88.15–96.56	51 (36.6%)	29.14–44.96	2 (1.4%)	0.4–5.09
p-value	4.9e−7		7e−		0		0		0		0.0053	

≤ 0.05 is taken as significance value

All SNPs detected in *pfdhfr* and *pfdhps* in the different years included in the study are described. Frequencies, 95% CIs and p-values are indicated

The most important increase detected for the ***pfdhps*** gene was in 437G, which increased significantly from a frequency of 50% in 1999 to 93.5% in 2019 (p = 0.000). The frequency of 540E also increased, from 6.6% in 1999 to 36.6% in 2019, but remains below the threshold frequency (50%) considered to indicate a lack of effectiveness of the SP in the IPT. In contrast, the frequency of the 581G mutation decreased from 5% in 1999 to 1.4% in 2019, although this decrease is not significant. The frequencies of the SNPs 540E and 581G in 2019 were 36.6 and 1.4%, respectively, values which are almost six times higher than that detected in 2016 for 540E but almost four times lower for 581G. The increase in frequencies over time was clear, with the exception of 581G, which decreased from 3% in 2016 to 1.4% in 2019; the frequency of each SNP detected in each of the years included in the study is detailed in Table 2.

#### Haplotypes *pfdhfr* + *pfdhps*

Combinations of the SNPs in both genes, *pfdhfr* and *pfdhps*, were classified into three haplotypes named partially resistant, fully resistant and super resistant [18] as described in Methods section. A partially resistant haplotype (IRNG) with a frequency of 43.3% appeared in 1999, subsequently increasing gradually over the time to reach a maximum of 86.3% in 2019 (Table 3). Significant differences can be observed when all years are compared (p < 0.001). The fully resistant (IRNGE) haplotype appears with a frequency of 8.3% in 1999, subsequently increasing to 11.2% in 2013 and 11.8% in 2016. However, this haplotype showed a sharp increase to 30.2% in 2019, an increase of almost three times the frequency detected in 2016 in only 3 years. Significant differences were detected between the different years, from 1999 to 2019 (p = 0; Table 3). The super resistant haplotype (IRNGEG) was not detected in 1999, 2001, 2006, 2011, or 2019 but was detected in 2004/5, 2013 and 2016 at a low frequency of 0.39, 0.6 and 1.7%, respectively (Table 3). Table 3 clearly shows that the frequencies of the different haplotypes have changed significantly over the years. Importantly, the super-resistant haplotype was not detected in 2019, so continued surveillance may ensure that SP remains useful for IPT.

**Table 3:** Frequency of haplotypes over time

Year (N)	Partially resistant (IRNG)		Fully resistant (IRNGE)		Super resistant (IRNGEG)	
	N (Freq.)	95% CI	N (Freq.)	95% CI	N (Freq.)	95% CI
1999 (60)	26 (43.3%)	31.57–55.9	5 (8.3%)	3.61–18.07	0	0–6.02
2001 (102)	44 (43.1%)	33.95–52.83	0	0–3.63	0	0–3.63
2004/5 (262)	149 (56.8%)	50.8–62.9	10 (3.9%)	2.09–6.88	1 (0.39%)	7e-02–2.13
2006 (158)	99 (62.6%)	54.9–69.8	3 (1.89%)	0.65–5.43	0	0–2.37
2011 (163)	130 (79.75%)	72.93–85.21	6 (3.7%)	1.70–7.80	0	0–2.3
2013 (170)	145 (85.2%)	79.19–89.84	19 (11.2%)	7.27–16.8	1 (0.6%)	0.10–3.26
2016 (169)	137 (81.1%)	74.49–86.26	20 (11.8%)	7.79–17.57	3 (1.7%)	0.61–5.09
2019 (139)	120 (86.3%)	79.64–91.07	42 (30.2%)	23.20–38.30	0	0–2.69
p-value	0		0		0.161	

≤ 0.05 is taken as significance value. N: number of samples analysed

The frequency of each of the three haplotypes into which they have been classified: partially resistant 51I/59R/108 N/437G (IRNG), fully resistant 51I/59R/108N/437G/540E (IRNGE) and super resistant 51I/59R/108N/437G/540E/581G (IRNGEG)

**Fig. 2:**
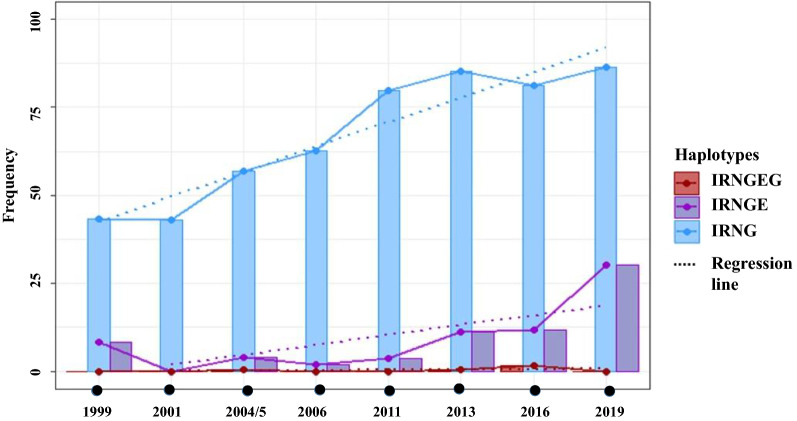
Evolution of haplotype frequencies over time. The upward trend of the partially (51I/59R/108N/437G, IRNG) and fully resistant (51I/59R/108N/437G/540E, IRNGE) haplotypes is shown over time, except for the super-resistant haplotype (51I/59R/108N/437G/540E/581G, IRNGEG), which is found to exhibit a downward trend. The regression line (dotted line) indicates the trends for each haplotype. The Figure highlights the different efficacy studies that have been carried out in Equatorial Guinea, as well as the changes in treatments until 2020

The logistic regression showed that, from 2001 to 2019, the probability of finding partially resistant haplotypes increased significantly in most years. This upward trend was confirmed using the Mann–Kendall trend test (tau = 0.857, p-value = 0.004). The odds of finding partially and fully resistant haplotypes was found to increase gradually over time, with the exception of 2016, reaching a maximum in 2019 (OR = 3.53 for IRNG and OR = 3.72 for IRNGE (Fig. 2).

The increase in the fully resistant haplotype in 2019 is at the expense of its partially resistant counterpart. This does not mean that there was no significant increase in new partially resistant cases, simply that these quadruple mutant parasites are acquiring the fifth mutation. Encouragingly, the super resistant haplotype remains at only a low frequency.

### Haplotypes in *pfmdr1* and *pfcrt*

All the SNPs related with resistance to CQ or AQ decreased in frequency from 1999 to 2019 (Table 4).

**Table 4:** Frequencies of SNPs and haplotypes in *pfmdr1* and *pfcrt* genes over time

Year (N)	*pfmdr1*						*pfcrt*		*pfmdr1* + *pfcrt*	
	86Y		1246Y		86Y/1246Y		76T		86Y/76T	
	N (%)	95% CI	N (%)	95% CI	N (%)	95% CI	N (%)	95% CI	N (%)	95% CI
1999 (60)	37 (61.6%)	49.02–72.91	4 (6.6%)	2.62–15.93	1 (1.6%)	0.29–8.86	43 (71.6%)	59.23–81.49	32 (53.3%)	40.89–65.37
2001 (102)	98 (96.1%)	90.35–98.46	24 (23.5%)	16.35–32.63	21 (20.5%)	13.88–29.43	87 (85.3%)	77.15–90.88	84 (82.3%)	73.82–88.54
2004/5 (262)	220 (83.9%)	79.04–87.92	3 (1.1%)	0.39–3.31	3 (1.1%)	0.39–3.31	200 (76.3%)	70.83–81.08	170 (64.8%)	59.93–70.41
2006 (158)	113 (71.5%)	64.04–77.98	1 (0.6%)	0.11–3.5	1 (0.6%)	0.11–3.5	107 (67.7%)	60.09–74.52	76 (48.1%)	40.45–55.84
2011 (163)	114 (69.9%)	62.51–76.45	0	0–2.30	0	0–2.30	114 (69.9%)	62.51–76.45	78 (47.8%)	40.32–55.48
2013 (170)	125 (73.5%)	66.43–79.59	3 (1.7%)	0.6–5.06	1 (0.6%)	0.10–3.26	109 (64.1%)	56.67–70.94	81 (47.6%)	40.27–55.12
2016 (169)	47 (27.8%)	21.61–35	0	0–2.22	0	0–2.22	33 (19.5%)	14.26–26.15	12 (7.1%)	4.11–12
2019 (n139)	19 (13.6%)	8.93–20.36	0	0–2.29	0	0–2.29	4 (2.8%)	1.12–7.17	0	0–2.29
p-value	0		0		0		0		0	

p-value ≤ 0.05 is taken as significance value. “No *ptmdr1*-86/*pfcrt*-76 double mutant detected in 2019, could be due to sample size”

Frequencies of SNPs detected in both *pfmdr1* and *pfcrt* genes, as well as frequencies of haplotypes both within *pfmdr1* (86Y/1246Y) and the combination of *pfmdr1* and pfcrt (86Y/76T) for each year included in the study

#### *Pfmdr1* gene

The frequency of the 86Y mutation was found to increase from 1999 to 2001, when it reached a maximum (96.1%), subsequently decreasing until 2019, when it was detected at a frequency of 13.6%. Significant differences were detected when comparing the evolution between 1999 and 2019 (p = 0.00). A similar evolution was detected for the other point mutation (1246Y), the frequency of which reached a maximum in 2001 (23.5%), subsequently decreasing until it could no longer be detected in 2016. The maximum frequency for the haplotype 86Y/1246Y (YY) was also seen in 2001 (20.5%), and this haplotype could also not be detected in 2016 (Table 4). The SNPs 86Y was still detected in 2019, although at a low frequency, whereas 1246Y was no longer detected in 2019.

#### *Pfcrt* gene

It is important to note that a frequency peak of the 76T was detected in 2001 in all cases, after which frequencies began to fall. The mutation 76T was still detected in 2019, although at a low frequency. The frequency of the mutation at this point in the gene (76T) was more frequent than mutations in *pfmdr1*, although their evolution was similar. Thus, it appears with a frequency of 71.6% in 1999, peaking in 2001 (85.3%), from where its presence decreased to a minimum of 2.8% in 2019 (Table 4).

**Fig. 3:**
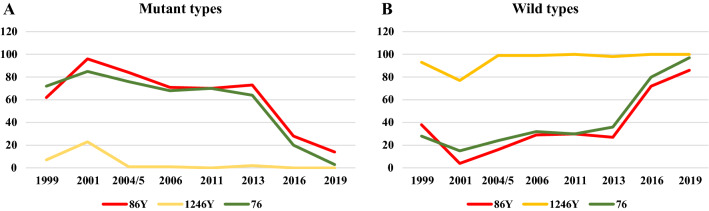
Evolution of mutant and wild types over time. Graph A shows how mutant populations are decreasing over time, while graph B shows how wild populations are increasing. Since the withdrawal of chloroquine (CQ) as a treatment, the selective pressure exerted by CQ is removed, so wild populations tend to increase over time

Many mutant populations are decreasing over time, while the wild populations are increasing, probably due to elimination of the selective pressure exerted by CQ since its withdrawal from use in 2001 (Fig. 3).

Similarly, the haplotype formed by the mutations in 86Y/76T (YT) increased from 1999 to 2001, when a maximum frequency of 82.3% was detected. From this point, its frequency decreased significantly until 2019, when it could no longer be detected (OR = 0.21, p-value < 0.05). The decreasing trend in resistant haplotypes of *pfmdr1*/*pfcrt* was confirmed using the Mann–Kendall trend test (tau = − 0.86, p-value = 0.016).

### Haplotypes in *pfk13*

#### Pfk13

Sequences for the K13-propeller domain were generated successfully for all the samples analysed in this study. Nearly all samples (1209/1223, 98.8%) were found to contain a wild-type allele. In contrast, SNPs were detected in only 13 samples (13/1223, 1.06%), and one sample contained two SNPs. As such, 13 samples contained a total of 14 SNPs. The frequency of samples containing mutations (synonymous and non-synonymous) over the years was 0.98% in 2001, 0.76% in 2004/5, 0.6% in 2006, 1.76% in 2013 and 5% in 2019 (Table 5).

**Table 5:** SNPs detected in the *pfk13* gene by sequencing

Year (N)	Sample ID	No codon	Wild allele		Mutant allele		Mutation	Type	Freq
			Sequence (nt)	AA	Sequence (nt)	AA			
2001 N = 102	136-H	612	ga**A**	Glu	ga**G**	Glu	E612E	S	0.98%
2004–5 N = 262	HQ-019	569	g**C**a	Ala	g**T**a	Val	A569V	NS	0.76%
	HFA-106	645	aa**C**	**A**sn	aa**T**	Asn	N645N	S	
2006 N = 158	101_9_3	663	**C**ta	Leu	**T**ta	Leu	L663L	S	0.6%
2013 N = 170	210.01.02	469	tg**C**	Cys	tg**T**	Cys	C469C	S	1.76%
	110.01.01	510	g**T**g	Val	g**C**g	Ala	V510A	NS	
	203.01.02	578	**G**ct	Ala	**T**ct	Ser	A578S	NS	
2019 N = 139	2974	532	t**G**t	Cys	t**A**t	Tyr	C532Y	NS	5.03%
		638	**G**ga	Gly	**A**ga	Arg	G638R	NS	
	3235	469	tg**C**	Cys	tg**T**	Cys	C469C	S	
	3502	544	**G**gg	Gly	**A**gg	Arg	G544R	NS	
	3756	469	tg**C**	Cys	tg**T**	Cys	C469C	S	
	3812	668	**G**ag	Glu	**A**ag	Lys	E668L	NS	
	3819	464	**G**at	Asp	**A**at	Asn	D3819N	NS	

SNPs detected in the *pfk13* gene after sequencing and their study. The type of mutation detected, synonymous or non-synonymous is indicated

ID: identification; AA: amino acid, nt: nucleotide; Freq.: frequency. A capital letter in the codon sequence indicates the muted nucleotide; S: Synonymous; NS: Non-Synonymous. The nucleotide that changes with the mutation is shown in capital letters and in bold

A total of 14 SNPs were detected, with six of these being synonymous (6/14; 42.8%) and eight non-synonymous (8/14; 57%). The six synonymous k13 mutations were found in positions G612G (2001), N645N (2004/5), L663L (2006), C469C (2013), and two C469C (2019). The eight non-synonymous mutations detected in *pfk13* gene were A569V in 2004/5, V510A and A578S in 2013, and C532Y, G638R, G544R, E668L, and D464N in 2019. The highest number of mutations was detected in 2019, and most of the mutations detected were non-synonymous. All mutations detected are shown in detail in Table 5.

**Fig. 4:**
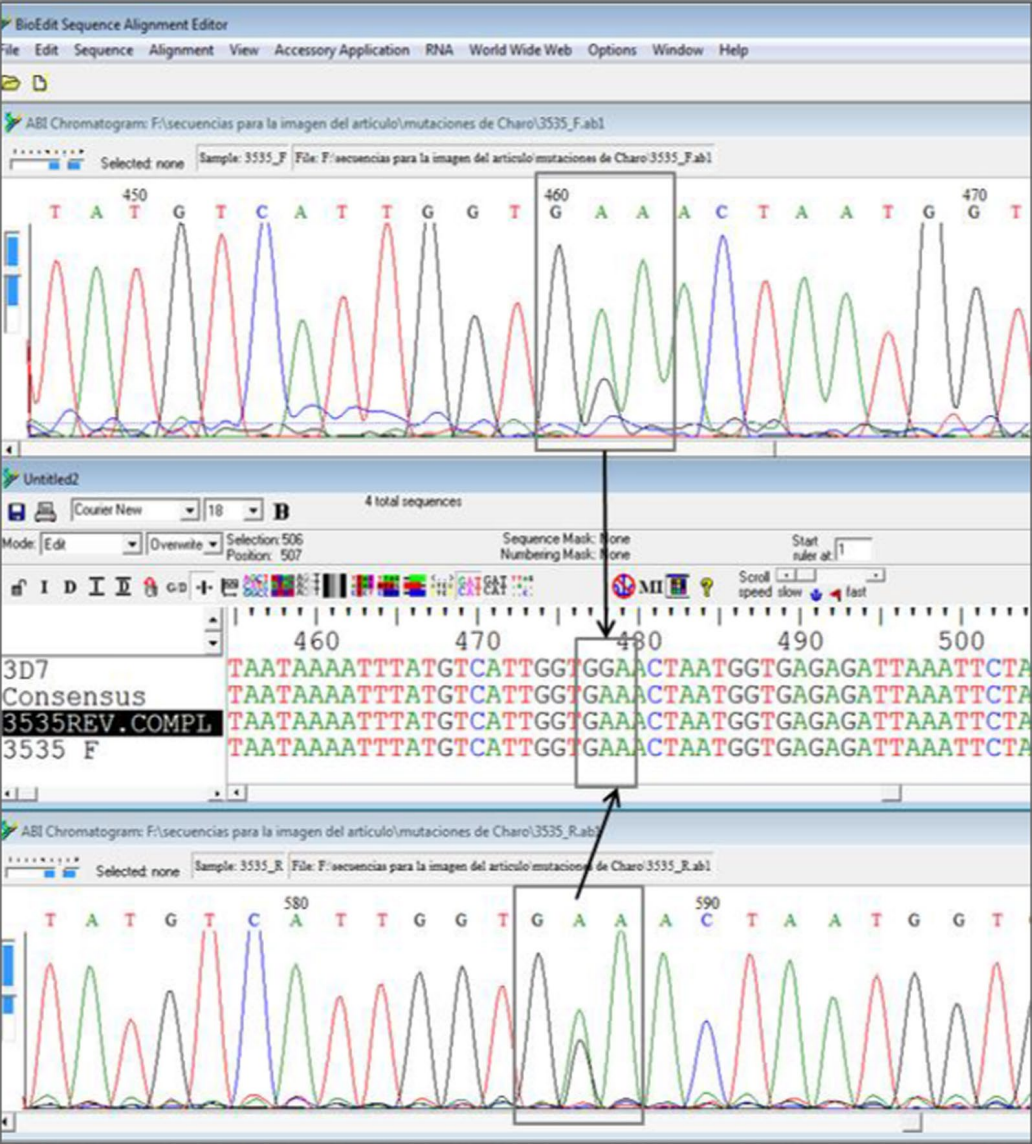
Comparison of forward and reverse sequences. The alignment and electropherograms of the forward and reverse complement sequences compared to the 3D7 sequence. The area where the mutation is detected is boxed. A peak corresponding to an A (mutant) is observed in both cases and below it, with almost the same intensity, a smaller peak corresponding to a G (wild). This could be therefore a mix of *P. falciparum* G**A**A (mutant population) and G**G**A (wild type population)

In a sample from 2019 in which a non-synonymous mutation (GGA to GAA; G592E) was detected, sequence analysis suggests that this could be a mixture of *P. falciparum* populations (wild and mutant parasites; Fig. 4).

## Discussion

A large number of SNPs related to resistance to different anti-malarial drugs that have been used as first-line or preventive treatment have been evaluated in this study. Evolution of resistance to SP (*pfdhfr*, *pfdhps*), CQ and AQ (*pfmdr1* and *pfcrt*) and artemisinin (*pfk13*) has been assessed taking into account the changes in public health treatment strategies.

Normally, when a country withdraws a given treatment due to drug resistance, the presence of sensitive parasites increases with respect to the resistant population over a given time period. However, it should also be borne in mind that misuse of treatments and the great plasticity of the parasite can make it possible for mutant parasites to increase and spread.

Numerous efficacy studies of anti-malarial drugs were carried out in Equatorial Guinea between 1999 and 2019. Moreover, first-line treatments changed over time until 2008, when ACT was introduced, the first being ASAQ. From 1990 to 1992, in vitro studies [44] found that CQ had a resistance of 16% and SP resistance was not particularly high (> 5%), meaning that SP could be used as a treatment in Equatorial Guinea. A subsequent in vivo study assessing the evolution of CQ and SP efficacy from 1992 to 1999 [45] found that CQ had 40% resistance and SP had 16% resistance, therefore CQ and SP were not used as treatment for malaria after 1999. Although CQ was withdrawn as a treatment in 1999, it can be seen that the frequency of resistance-related mutations in *pfmd1*, *pfcrt* and the combination *pfmdr1* + *pfcrt* reached a maximum in 2001, after which it started to decrease. This may be because it is estimated to usually take 2 years for the parasite to show any change in response to treatment, which is why efficacy studies are conducted at least every 2 years. However, this may also be explained by the delay in implementing a uniform treatment strategy throughout the country. Resistance to CQ is linked to a mutation at codon 76 of *pfcrt*, which has proven to be the chief determinant of CQ resistance [46]. As such, the decreasing trend in this mutation and the haplotype 86Y/76 T indicates that the withdrawal of CQ as a treatment was effective and has been maintained over time. As the selective pressure exerted by CQ on the parasite population has been removed, the frequency of the wild-type population has increased with respect to the mutant population. As noted by Achieng et al. [47], what would be expected over time is a greater increase of wild *pfcrt* K76 and *pfmdr1* N86 due to the longer withdrawal time of CQ and distribution of AL as treatment. It would therefore be of interest to revisit the mutational profile for the *pfmdr1* and *pfcrt* genes of *P. falciparum* over a period of at least 2 years to see how this changed in the presence of AL.

The downward trend in these mutations means that the susceptible population is higher than the mutant population, thus meaning that the efficacy of other anti-malarial drugs such as AQ and MQ is assured. However, other studies have shown that the re-introduction of CQ treatment might rapidly lead to the selection of mutant populations again [48].

Likewise, selection of the wild-type K76 codon in the *P. falciparum* CQ resistance transporter gene (*pfcrt*) has been associated with pressure from AL treatment (first-line of treatment in Equatorial Guinea from 2020) in a number of studies [49–54]. Indeed, dramatic increases in the prevalence of wild-type *pfcrt* K76 and *pfmdr1* N86 have been associated with discontinuation of CQ and deployment of AL in western Kenya, although AL continued to be effective with these changes [47]. In Equatorial Guinea, there has been an evolution in this direction since 1999, i.e., a decrease in mutants in favour of an increase in wild populations. Thus, the wild genotype *pfmdr1* N86 had a frequency of 86%, whereas that for *pfcrt* K76 was 100%, in 2019.

Although it was known from previous studies that there was 25% resistance to SP in Equatorial Guinea since 1999 [45], it was still used as a treatment, either alone or in combination, until 2008. In 2005 [3] a new study in which the combinations ASSP and ASAQ were tested was carried out in order to have two treatment alternatives in combination. Despite its slightly yet constantly increasing trend in resistance, SP was never discontinued as a treatment. These combinations were used in Equatorial Guinea from 2003 until 2008, when ASAQ, the first ACT used in that country, was introduced. The use of SP in combination with AQ and AS has been widespread for many years, with serious harm to resistance that its use entails. Accordingly, this has led to an increase in genetically resistant populations, as can be seen in the Results section, and some SNPs can be seen that are fixed in the population, as is the case of *pfdhfr* 108N, which has a frequency of 100%. The use of these combinations has created a scenario with selective pressure in which some of the mutations not only increase and spread but also become fixed in the parasite population.

Importantly, the 164L mutation in *pfdhfr*, which is related to a significant resistance to SP [55], has not been detected in any of the years studied in this study. However, this mutation was previously detected in Equatorial Guinea, in a previously published article with samples from 2013 [56]. In light of the above, data obtained from the analysis of *pfdhfr* + *pfdhps* haplotypes over time reveal that there has been no real or total withdrawal of SP as a treatment. It can be seen that the partially resistant (IRNG) and fully resistant (IRNGE) haplotypes have been progressively increasing since 1999, reaching their peak in 2019, whereas the super-resistant (IRNGEG) have always had a low trend over time, never exceeding 2% and not being found in 2019. The data obtained are consistent with previous studies with samples from the island of Bioko, where the partially and fully resistant types were the most common and the frequency of the super-resistant type was very low [57].

Evidence for the misuse of SP in the mainland region of Equatorial Guinea, where it was found that 27.3% of children had received artemether in monotherapy, 13.8% SP and only 6.8% had received the official ACT (ASAQ) [58], supports the hypothesis of the influence of incorrect treatments on the evolution of resistance. This is probably because the official first- and second-line treatments are not available countrywide. As SP is still used as a treatment and has not been reserved only for SP-IPT in the two main populations vulnerable to malaria, namely pregnant women (SP-IPTp) and children under 5 years of age (SP-IPTi), its efficacy has been compromised [17, 59].

Although resistance-related haplotypes to SP exhibit an upward trend, it has been observed that mutations at positions 540E (36.6%) and 581G (1.4%) are not sufficiently high to jeopardize the use of SP in IPT. Taking into account the results, it can be inferred that the increase of the fully resistant haplotype in 2019 is at the expense of its partially resistant counterpart. This means that there was no significant increase in new partially resistant cases, simply that these quadruple mutant parasites are acquiring a fifth mutation.

Current WHO recommendations suggest that SP-IPTp should be discontinued if the frequency of 540E exceeds 50% and that of 581G exceeds 10% [57]. Based on current evidence, IPTp and IPTi remain effective in preventing the adverse consequences of malaria on maternal, foetal and infant outcomes in Equatorial Guinea. However, the implementation of control measures in the country should be maintained to avoid the spread of these mutations and the consequent reduction in the efficacy of IPTp.

The data obtained in this study are similar to those observed in countries bordering Equatorial Guinea, such as Cameroon and Gabon. In Gabon [60], for instance, the partially resistant haplotype appeared in 2014 with a frequency of 92.9% (compared with 85.2 and 81.1% in Equatorial Guinea in 2013 and 2016, respectively). Similarly, this haplotype is also the most frequent in Cameroon [61]. Therefore, it seems that the distribution of parasites with resistant haplotypes to SP is quite homogeneous in the area.

The introduction of ACT as a treatment for malaria was very effective in mitigating the threat of resistance to anti-malarial treatments. In 2006 [5], an efficacy study was carried out to determine the efficacy of ASAQ, the first time that an ACT for treating uncomplicated malaria had been tested in Equatorial Guinea. As a result, ASAQ began to be used as first-line treatment in 2008, when the National Guidelines were changed, and AL as second line. Two years after the introduction of ACT, in 2010, a new efficacy study was conducted for ASAQ [62] and its efficacy was found to be 95%, therefore its use as first-line treatment was maintained. However, it was difficult to maintain patient adherence [63] to this treatment due to side effects such as headache, nausea, tinnitus, and fatigue. The presence of these side effects, and the lack of adherence to treatment, has led to the use of artemether as monotherapy in some areas of the country. Monotherapy is not permitted by the WHO because it may favour the emergence of ART-resistant parasite populations, which could threaten the future efficacy of ACT in the country.

The last efficacy study carried out in Equatorial Guinea, in 2017/2018 [6], showed that the efficacy of ASAQ and AL was close to 95% and that no ART resistance was detected. Following the completion of this efficacy study, a new National Therapeutic Guideline for malaria was published in January 2020. This new guideline shows a change in the lines of treatment, recommending AL as the first line of treatment and ASAQ as the second, in order to facilitate patient adherence to treatment.

The study of *pfk13* gene sequences carried out herein to determine the presence of mutations related to resistance to ART, and therefore to ACT, allows us to ascertain whether this combination therapy is being used correctly in Equatorial Guinea. The development and spread of ART-resistant *P. falciparum* outside the Greater Mekong Sub-region (GMS) poses a great challenge, particularly to sub-Saharan Africa, where in 2020 it accounted for 90% of global malaria cases and 95% of malaria deaths [1]. Genetic analysis of the whole genome sequences previously performed showed that the resistant isolates were classified as an African-specific group. This suggests that they may have originated in Africa and not through the migration process from GMS [64].

Current data from this study reports a low prevalence (5%) for *pfk13* mutations, both synonymous and non-synonymous, and none of these was among those associated with ART clearance delay in Southeast Asia. The allelic frequencies reported for Central, West and East Africa are generally less than 6% [65–67]. The current study result is within this limit, because the frequency of the *pfk13* mutation in Equatorial Guinea has increased in relative terms since 1999, reaching 5% in 2019. A study conducted in Cameroon, a country bordering Equatorial Guinea, revealed a high mutation rate of 15.1% for isolates containing at least one non-synonymous mutation [68].

The most common non-synonymous mutation (A578S) observed in Africa was detected in a sample from 2013, as well as in two samples from the therapeutic study carried out in Equatorial Guinea (2017–2018) [6]. A similar study of *pfk13* carried out in Equatorial Guinea detected that 2.04% of cases exhibited the non-synonymous A578S mutation [69]. The same mutation was detected in the same year (2013) in Cameroon and Gabon [32, 60], both of which border the mainland region of Equatorial Guinea. This mutation (578S) was detected in the 2017/18 efficacy study but not in the 2019 samples in the current study [6]. Moreover, it is the most common mutation in Africa, therefore it is likely that if a larger number of samples from 2019 were analysed it would also be detected. The non-synonymous mutation E612K (**G**AA to **A**AA) was detected in Cameroon in 2017 [37] and the same mutation appeared in the current study, but synonymous (E612E, GA**A** to GA**G**), in a sample from 2001. Given that this mutation has been detected as non-synonymous in Cameroon and as synonymous in this study, it could be hypothesized that this point is an area of genetic instability. It could also indicate that this is the first step for a non-synonymous mutation to occur in Equatorial Guinea in the future. One interesting finding is the detection of the synonymous mutation C469C, which appears in one sample from 2013 and in two samples from 2019. It will be interesting to continue characterizing more isolates and to see if this mutation continues to appear, or if its frequency continues to increase over time. Surveillance will have to be established to see if in the future such a synonymous mutation could become non-synonymous and have clinical significance for ACT resistance.

It is essential to continue to make correct use of the first- and second-line treatments (AL and ASAQ, respectively) to avoid the appearance of new mutations, and good surveillance is essential to be able to quickly detect possible mutations from SEA that might be introduced into the country and, if they appear, to prevent them from spreading.

Taken together, the low frequencies of *pfk13* mutant alleles found in Equatorial Guinea suggest that ART-resistant parasites are not under evolutionary selection in this country, reinforcing the assumption that such mutations are rare in Africa. Furthermore, none of the polymorphisms known to be involved in ART resistance in Asia has been associated with ART resistance in Africa. Therefore, local ART-resistant *P. falciparum* strains may emerge independently in Equatorial Guinea and in the African continent under constant drug pressure from ACT, possible misuse of these drugs if treatment guidelines are not followed, non-adherence to treatment, self-medication and the introduction of counterfeit drugs as is known to be occurring [2].

The effect observed on the evolution of parasites with mutations related to CQ resistance, which have decreased significantly compared to parasites from 20 years ago, indicates that avoiding pharmacological pressure by withdrawing treatment is one of the most important aspects affecting the increase of sensitive parasites compared to resistant parasites. Regarding to mutations in *pfdhfr* and *pfdhps* is important to establish intensive surveillance because the use of SP as a preventive treatment in pregnant women and children under 5 years of age could be at risk. As for ACT, as recommended by WHO, treatments should only be administered when the presence of the parasite has been identified by a diagnostic method, avoiding unnecessary treatments. Compliance with the National Therapeutic Guidelines for malaria is mandatory to avoid the use of other treatments that have already been withdrawn and are no longer effective.

All of the above highlights the need for constant surveillance to detect resistance-related mutations early to prevent them from spreading. Consequently, this will ensure that the population receives better healthcare and that treatment to cure malaria is adequate, as a complete cure is a benefit not only for the patient, but also for the whole community.

## Conclusions

The study of resistance markers allows us to evaluate the efficacy of treatments, and to determine whether there is good adherence to them. These markers also allow us to evaluate how different public health strategies affect the parasite population.

The withdrawal of CQ as a treatment in Equatorial Guinea has been effective over time, as wild-type parasite populations outnumber mutant populations. The 86Y/76T haplotype (*pfmdr1* + *pfcrt*) has declined and could no longer be detected in 2019. The upward trend observed in SP resistance markers evidence its misuse, either alone or in combination with AS or AQ, in some areas of the country, although it should not have been used as a treatment for a long time now. This keeps the selective pressure of SP in the area, allowing partially and fully SP-resistant haplotypes to be very high. Although, super-resistant haplotypes are not yet found at a high frequency, without surveillance they could start to become more prevalent in the population.

As for the *pfk13* gene, it can be seen that since the incorporation of ACT as first-line treatment in 2008, no mutations have been detected in relation to ART resistance. However, it can be seen that there is a greater accumulation of non-synonymous mutations in 2019 compared to years prior to 2008, although these appear to be unrelated to resistance. Taking into account the results of the latest therapeutic efficacy study carried out in Equatorial Guinea (2018), ASAQ and AL have been shown to have a high efficacy for the treatment of uncomplicated malaria.

In light of all the data obtained, the National Malaria Programme must monitor the use of SP in order to reserve it exclusively for IPT, making its use mandatory and with supervision by the first- and second-line malaria treatment programme. To this end, there should be an equal and homogeneous distribution throughout the country, therapeutic efficacy studies every 2 years, and *pfk13* mutational profiling studies to detect possible resistance-related mutations in order to control their spread to other regions. Finally, rapid diagnosis and effective treatment are the basis for malaria control and are therefore essential for ensuring quality healthcare.

## Data Availability

The datasets used and/or analysed during the current study are available from the corresponding author on reasonable request.

## Abbreviations

AL: Artemether/lumefantrine
ART: Artemisinin
ACT: Artemisinin-based combination therapy
ASAQ: Artesunate-amodiaquine
BLAST: Basic Local Alignment Search Tool
CQ: Chloroquine
*pfcrt*: Chloroquine resistant transporter gene
pfdhfr: Dihydrofolate reductase gene
*pfdhps*: Dihydropteroate synthase gene
IRNGE: Fully resistant
IPT: Intermittent preventive treatment
IPTi: Intermittent preventive treatment in infants
IPTp: Intermittent preventive treatment in pregnant woman
*pfk13*: Kelch propeller domain on chromosome 13
L: Lumefantrine
MQ: Mefloquine
NM-PCR: Nested Multiplex Malaria PCR
*pfmdr1*: *P. falciparum* Multi-drug resistance gene
IRNG: Partially resistant
Q: Quinine
RFLP: Restriction-fragment length polymorphism
SNPs: Single Nucleotide Polymorphism
SEA: Southeast Asia
SP: Sulfadoxine/pyrimethamine
IRNGEG: Super resistant
WHO: World Health Organization
